# Prevalence, Predictors, and Prognosis of Depression After Transient Ischemic Attack: A Population-Based Study

**DOI:** 10.1161/STROKEAHA.125.052251

**Published:** 2025-11-20

**Authors:** Aubretia J. McColl, Ramon Luengo-Fernandez, Emily-Rose Vaughan-Fowler, Matthew B. Downer, Sarah T. Pendlebury, Lucy E. Binney, Louise E. Silver, Peter M. Rothwell

**Affiliations:** 1 Nuffield Department of Clinical Neurosciences, Wolfson Centre for the Prevention of Stroke and Dementia, Wolfson Building-John Radcliffe Hospital, University of Oxford, United Kingdom.

**Keywords:** cerebral infarction, depression, ischemic attack, transient, prognosis, stroke

## Abstract

**BACKGROUND:**

Depression is common after stroke and is associated with increased mortality. However, there are few data on the prevalence, predictors, or prognosis of depression after transient ischemic attack (TIA). Although poststroke depression is often assumed to be due to the brain lesion or disability, patients with TIA may also be susceptible due to shared risk factors, lifestyle/medication changes, or worries about stroke.

**METHODS:**

The prevalence of depression 1 and 12 months after TIA was assessed in a population-based cohort (OXVASC [Oxford Vascular Study]) from 2014 to 2020. Depression was related to risk factors (including infarction on brain imaging) in a multivariable logistic regression model and outcome (disability, quality of life, institutionalization, recurrent events, and mortality) in a Cox proportional hazard regression model during 5-year follow-up after adjustment for covariates that were found to be significant (*P*<0.1) in the age-/sex-adjusted models.

**RESULTS:**

Of 519 patients (mean age, 70.5 [13.1]; female, 51.1%), 126 (24.3%) had depression after TIA, with a higher rate at 1 versus 12 months (99/20.7% versus 66/14.9%; *P*=0.022). Depression was independently associated with younger age (adjusted odds ratio/decade, 0.74 [95% CI, 0.60–0.90]), low baseline mood (4.06 [95% CI, 2.31–7.15]), past depression (1.81 [95% CI, 1.09–3.03]), multimorbidity (1.19 [95% CI, 1.02–1.39]), living alone (1.94 [95% CI, 1.14–3.32]), disability (3.53 [95% CI, 1.89–6.59]), and deprivation (1.28 [95% CI, 1.03–1.59]). After adjustment for confounders, depression did not predict risk of recurrent vascular events (adjusted hazard ratio [aHR], 1.42 [95% CI, 0.76–2.64]; *P*=0.27) but did predict increased 5-year all-cause mortality (aHR, 2.27 [95% CI, 1.21–4.27]; *P*=0.011), independently of acute lesions on brain imaging (aHR, 2.18 [95% CI, 1.09–4.34]; *P*=0.027), and particularly when persistent (present at 1- and 12-month follow-up; aHR, 4.58 [95% CI, 2.07–10.13]; *P*<0.001). Persistent depression was also independently associated with disability (adjusted odds ratio, 12.10 [95% CI, 6.18–23.7]; *P*<0.001) and institutionalization (aHR, 5.83 [95% CI, 1.84–18.50]; *P*=0.003) within 5 years and with reduced quality of life (coefficient, −0.245; *P*<0.001).

**CONCLUSIONS:**

Depression is common early after TIA, independent of acute ischemia on brain imaging, and persistent depression is also strongly associated with adverse outcomes.

Major stroke can lead to a depressive disorder with a prevalence rate of around 30% in the first year, and though the mechanism(s) is not fully understood, it is associated with increased short- and long-term mortalities.^1–3^ Data on depression after a transient ischemic attack (TIA) are sparse, with only a few small studies reporting a prevalence of 10% to 20% during the first year after TIA,^4^ and no published data on prognosis,^5–9^ perhaps partly because patients with TIA are generally not screened for depression in routine practice. Yet, patients with TIA may also be at risk of depression due to new medications, lifestyle adjustments, worries about stroke risk, or possibly due to shared etiological factors, such as inflammation or sympathetic/adrenocortical activation,^10–13^ or even to immune mechanisms triggered by small cerebral infarcts.^14^

A better understanding of the prevalence, predictors, and prognosis of depression after TIA would be helpful in guiding clinical practice, including the need for routine screening during follow-up and potential role of antidepressant medication,^15^ and might also provide insights into the mechanism(s) of poststroke depression. For example, a high rate of depression after TIA cannot be related to new disability and would be unlikely to be a consequence of cerebral injury in patients without acute infarction on diffusion-weighted brain imaging but might implicate medication changes or underlying risk factors (eg, hypertension or smoking), which can themselves be associated with depression.^16,17^

In a large longitudinal population-based study, we, therefore, aimed to determine the prevalence of depression in the first year after a TIA in relation to potential predictors, including infarction on brain imaging, and to determine prognosis, including any impact on the risk of recurrent vascular events, quality of life (QoL), disability, and all-cause mortality during 5-year follow-up.

## Methods

Consecutive patients with a first-in-study TIA were recruited into the OXVASC (Oxford Vascular Study) from July 2014 (when the Geriatric Depression Scale was introduced into the assessments). Inclusion in this study was stopped in March 2020 due to the impact of the COVID-19 pandemic on recruitment and completion of face-to-face assessments. OXVASC is a longitudinal, prospective population-based study of the incidence and outcomes of all acute vascular events in a subpopulation of Oxfordshire, United Kingdom. The study population comprises all individuals (mid study mean, 94 567) registered with about 100 general practitioners in 8 general practices in Oxfordshire, United Kingdom. Ascertainment of patients, which began in April 2002, is through both hot and cold methods, which have been previously described and have been demonstrated to achieve near complete ascertainment of all acute vascular events.^18,19^ In brief, this includes direct referral to the study from participating general practices, daily screening of all admissions to an acute hospital, and emergency department attendances, regular review of brain and vascular imaging reports, regular review of general practice computer systems for patients coded with a cerebrovascular diagnosis, and reviews of death certificates and *International Classification of Diseases*, *Tenth Revision* vascular codes via public health, general practitioners, or the coroner’s office.

All patients with a TIA were reviewed by a study physician as soon as possible (usually within 1–2 days of seeking medical attention) after their initial presentation either on a hospital ward or in an outpatient neurovascular clinic. Patients gave informed consent to participate, or assent was obtained from a relative if a patient lacked capacity. A structured interview, examination, and brain imaging were completed, and all primary and secondary care medical records were reviewed.

Patients were asked at baseline about a prior or current diagnosis of depression. A history of depression was defined as a composite of self-reported history from the structured interview, coding in their primary care record, or pre-event use of antidepressant medication at a therapeutic dose according to guidelines.^20^ Patients were also asked at baseline whether they currently often feel sad or depressed to indicate a current low mood. Socioeconomic deprivation was defined by the index of multiple deprivation. Low education was defined as leaving school aged ≤16 years without any subsequent formal education. Vascular risk factors were defined by self-report at baseline but were verified against primary and secondary care records and baseline medication history. Severity of white matter disease was defined according to a modified Fazekas scale (for magnetic resonance imaging) and Age-Related White Matter Changes scale (for computed tomography), completed by a senior study neuroradiologist blinded to the clinical data. Acute lesions on brain imaging were also identified by the senior study neuroradiologist, blinded to the clinical data.

TIA and stroke were defined according to the National Institutes of Health criteria^21^ but also stratified according to the presence of an acute lesion on diffusion-weighted magnetic resonance brain imaging. The senior study neurologist (P.M.R.) reviewed all cases to confirm diagnosis and management.

As part of the baseline assessment, patients were asked if they currently often feel sad or depressed. At face-to-face follow-up at 1 and 12 months, a structured interview including the Geriatric Depression Scale (GDS) was administered by a study nurse or physician, and use of antidepressant medication was recorded. Depression was defined as a GDS score 5 or above at either the 1- or 12-month assessment.^22^ The GDS has been validated in both a stroke and TIA population.^23^ To minimize attrition, if a patient was unable to attend the hospital for their follow-up, then a home visit was completed instead, and some patients had telephone follow-up assessments during the COVID-19 pandemic.

When depression was identified at follow-up, a letter was written to the patient’s primary care physician informing them of this finding. A recurrent vascular event was defined as a TIA, ischemic stroke, subarachnoid hemorrhage, intracerebral hemorrhage, acute cardiac events (myocardial infarction and sudden cardiac death), and peripheral vascular events. Deaths were recorded by review of both primary care and hospital records, access to centralized UK death records, and continued day-to-day case ascertainment.

The study was conducted according to the STROBE statement (Strengthening the Reporting of Observational Studies in Epidemiology).^24^

### Statistical Analysis

Prevalence rates of depression after a TIA were calculated at baseline, 1 month, and 12 months. Patients who completed 1 month and then also 12 months of follow-up were compared with those with incomplete follow-up. Continuous variables were compared by the Student *t* test and categorical variables by the X^2^ test. Statistical significance was defined as *P*<0.05. Age-/sex-adjusted associations between baseline characteristics and postevent depression (at 1 month, at 12 months, and at either 1 or 12 months) were determined by binary logistic regression. Variables that were significant (*P*<0.1) in the age-/sex-adjusted univariable analysis were included in the multivariable regression analysis. Age and sex were also included in the multivariable regression analysis as they have been found to be associated with depression in prior studies.^5,6,25^ A sensitivity analysis was performed excluding patients who had either a history of depression or had reported low mood at the initial assessment after their TIA.

Cox proportional hazard models were used to relate depression to the risk of recurrent vascular events and all-cause death during follow-up, with censoring at January 2024, death, or 5-year follow-up. Crude, age-/sex-adjusted, and multivariable adjusted hazard ratios were determined for each outcome. Variables that were significant (*P*<0.1) in the age-/sex-adjusted univariable analysis were included in the multivariable regression analysis. For all-cause death, separate sensitivity analyses were done excluding patients who had a history of depression, those on antidepressant medication at any point during the initial 12 months, or who had an acute vascular lesion on baseline brain imaging. Kaplan-Meier curves were plotted for the risk of death by the presence of depression at a single assessment, persistent, or no depression, and statistical significance of differences was assessed with log-rank tests.

Disability was defined at a modified Rankin Scale score of ≥2 at baseline or within 5 years of follow-up. Health-related QoL was assessed using the Euroqol 5-Dimensions 3-Levels (http://www.euroqol.org). Patients report problems (none, some, or unable/extreme) in 5 attributes (mobility, usual activities, self-care, anxiety/depression, and pain). Responses were converted into utilities using UK tariffs ranging from −0.59 (worse than death) to 1 (full health).^26^ The Euroqol 5-Dimensions has been validated for stroke/TIA.^27^ Time to institutionalization was defined as the difference between the date on which the TIA occurred and the date of admission into a nursing or residential care home. Five-year survival to institutionalization was estimated using the Kaplan-Meier survival function, adjusted for censoring, and using a Cox proportional hazard regression model. Patients who were in a care home pre-event were excluded from the analysis. To assess whether postevent depression was associated with disability or QoL at any point during the 5-year follow-up, multivariable logistic (for disability) and ordinary least squares (for utility) regression analyses for panel data modeling using random effects models were performed.^28^ Random effects models were chosen, given that all our independent variables were time invariant. In addition, the random effects’ specification gives a comprehensive model because it uses a weighted average of the between-patients and the within-patients (over time) estimations. Analyzing utility using ordinary least squares with robust standard errors has been shown to be valid and provides unbiased estimates.^29^ Variables that were significant (*P*<0.1) in the age-/sex-adjusted logistic regression analysis of post-TIA depression were included as covariables. Statistical analyses were performed using SPSS Statistics 25.

### Data Availability

Requests for access to data should be submitted for consideration to the OXVASC Study Director (peter.rothwell@ndcn.ox.ac.uk).

## Results

Among 562 patients with a first-in-study TIA, 519 (92.3%) had at least 1 follow-up GDS assessment (478 at 1 month and 442 at 12 months; Figure S1). Reasons for non-follow-up and patient characteristics are given in Tables S1 and S2. Patients who did not complete the 1-month follow-up were more likely to be female (61.9% versus 50.0%; *P*=0.044) and had higher levels of premorbid disability (modified Rankin Scale score ≥2: 33.3% versus 15.9%; *P*<0.001) than those who did. Patients who did not complete the 12-month follow-up were more likely to have atrial fibrillation (18.2% versus 9.5%; *P*=0.025) and higher levels of premorbid disability (29.9% versus 13.2%; *P*<0.001) than those who did.

### Prevalence

Of the 519 patients who had a GDS assessment, 126 (24.3%) had depression at either 1 or 12 months, with a higher rate in those seen at 1 versus 12 months (99/478; 20.7% versus 66/442; 14.9%; *P*=0.022; mean GDS score [SD], 2.8 (2.9) versus 2.3 (2.8); *P*=0.012) overall, with a consistent trend in the 401 patients who completed both assessments (79/19.7% versus 57/14.2%; *P*=0.038; mean score, 2.7 [2.9] versus 2.2 [2.6]; *P*=0.008; Figure S2).

### Predictors

On multivariable analysis, any depression within the first 12 months was associated with younger age (adjusted odds ratio/decade, 0.74 [95% CI, 0.60–0.90]; *P*=0.004), low mood at baseline (4.06 [95% CI, 2.31–7.14]; *P*<0.001), history of depression (1.81 [95% CI, 1.09–3.03]; *P*=0.023), Charlson Comorbidity Index (1.19/point [95% CI, 1.02–1.39]; *P*=0.028), modified Rankin Scale score ≥2 (3.53 [95% CI, 1.89–6.59]; *P*<0.001), living alone (1.94 [95% CI, 1.14–3.32]; *P*=0.015), and socioeconomic deprivation (1.28 per SD of index of multiple deprivation score [95% CI, 1.03–1.59]; *P*=0.025). Depression was not associated with the duration of TIA symptoms or the presence of an acute lesion on brain imaging (Table 1). Associations were broadly similar for depression that was present at both the 1- and 12-month assessments (Table S3).

**Table 1.**
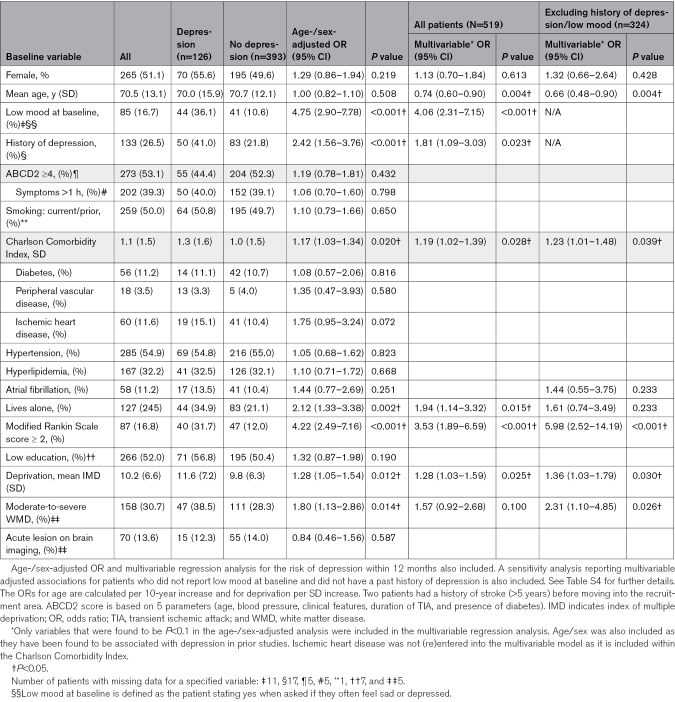
Associations Between Baseline Characteristics of Patients With TIA and Their Risk of Depression Within the Subsequent 12 Months for All Patients (N=519)

Among 324 patients without a history of depression or low mood reported at baseline, 53 (16.4%) developed depression within the first 12 months. Depression was again associated with younger age (adjusted odds ratio, 0.66 per decade [95% CI, 0.48–0.90]; *P*=0.004), higher Charlson Comorbidity Index (1.23 per point [95% CI, 1.01–1.48]; *P*=0.039), modified Rankin Scale score ≥2 (5.98 [95% CI, 2.52–14.19]; *P*<0.001), higher socioeconomic deprivation (1.36 [95% CI, 1.03–1.79]; *P*=0.030), and white matter disease on brain imaging (2.31 [95% CI, 1.10–4.85]; *P*=0.026; Table 1; Table S4).

### Prognosis

**Figure 1.**
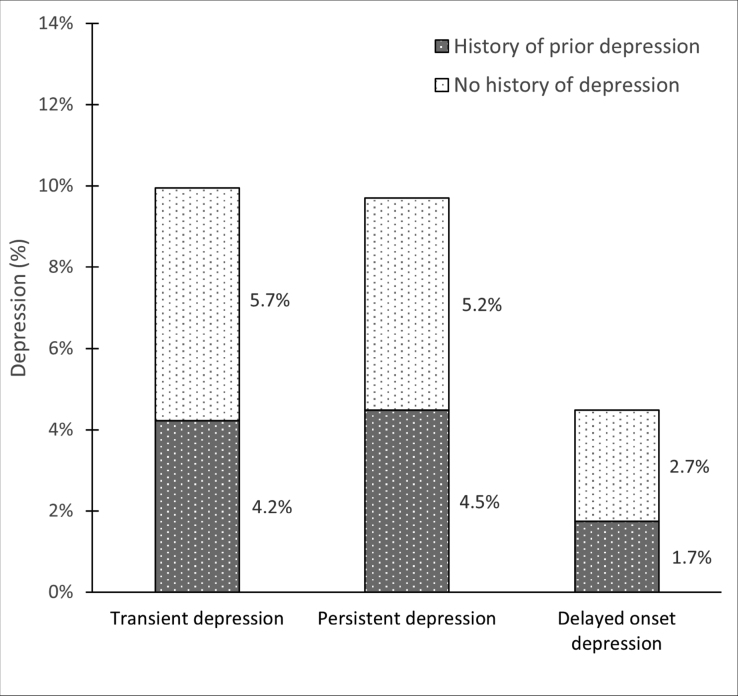
**Proportion of patients with persistent, transient, or delayed-onset depression after a transient ischemic attack.** Patients who completed both the 1- and 12-month assessments were included. Persistent depression is defined as present at both 1 and 12 months. Transient is defined as present at 1 month but not at 12 months. Delayed is defined as present at 12 months but not at 1 month. Patients are stratified by the presence or absence of a history of depression or low mood at baseline. The rates of persistent, transient, and delayed-onset depression were broadly similar for the cohort who completed their 1- and 12-month assessments before the COVID-19 pandemic (10.2%/9.9%/4.8%, n=333) compared with those who completed their assessments after the start of the COVID-19 pandemic (7.4%/10.3%/2.9%, n=68).

Among the 401 patients assessed at both 1 and 12 months, 39 (49.4%) of the 79 who were depressed at 1 month remained depressed at 12 months, and a further 18 (31.6%) had developed new depression at 12 months (Figure 1). Depression at 1 month was strongly predictive of depression at 12 months (age-/sex-adjusted odds ratio, 17.27 [95% CI, 8.93–33.38]; *P*<0.001). Depression at 1 month did not predict the risk of recurrent vascular events at 12 months (adjusted hazard ratio [aHR], 1.35 [95% CI, 0.60–3.02]; *P*=0.47).

**Figure 2.**
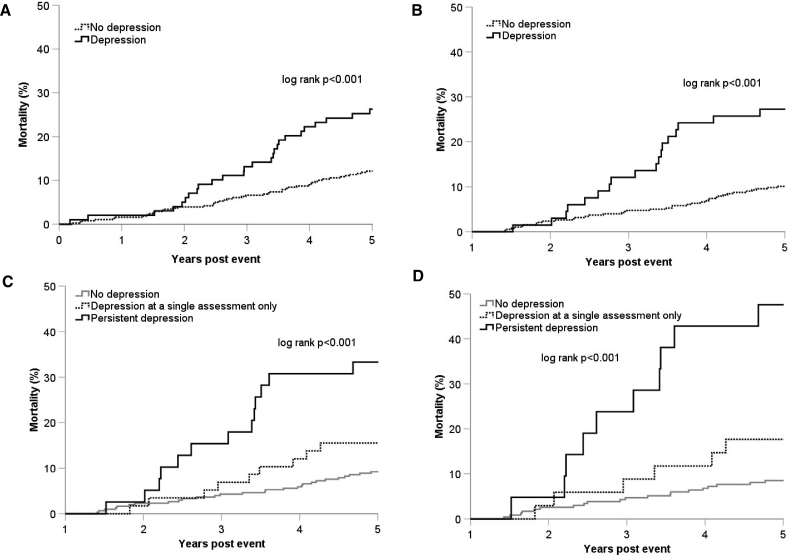
**Risk of (any) death after a TIA stratified by the presence, or absence, of depression.** Stratified by the presence of (**A**) depression at 1 month (n=478), (**B**) depression at 12 months (n=442), (**C**) depression at a single assessment, persistent, or no depression after a transient ischemic attack (TIA; n=401), and (**D**) depression at a single assessment, persistent, or no depression after a TIA in patients excluding those with a past medical history of depression (n=290). Patients who died before (**A**) 1- or (**B**–**D**) 12-month assessments were excluded.

Neither depression at a single assessment (aHR, 1.47 [95% CI, 0.76–2.86]; *P*=0.26) nor depression at both assessments (1.30 [95% CI, 0.55–3.07]; *P*=0.55) predicted the 5-year risk of any acute vascular event (n=60; Table 2). Although interpretation is limited due to the small number of events, persistent depression (ie, at both assessments) was associated with increased risk of acute cardiac events (aHR, 32.17 [95% CI, 3.24–319.37]; *P*=0.003), but there were no strokes during follow-up in this subgroup (Table 2). Moreover, although no deaths were due to suicide (Table S5), depression at 1 month and depression at 12 months were both associated with increased 5-year mortality (*P*<0.001; Figure 2), particularly for persistent depression (aHR, 4.58 [95% CI, 2.07–10.13]; *P*<0.001; Figure 2; Table S6). This relationship was present for those without an acute brain lesion on imaging (aHR, 4.62 [95% CI, 1.94–11.01]; *P*<0.001), those with no premorbid history of depression (aHR, 8.03 [95% CI, 2.95–21.85]; *P*<0.001; Figure 2), and those on no antidepressant medication (aHR, 5.39 [95% CI, 2.18–13.32]; *P*<0.001; Table S6). Antidepressant use was low in patients with depression at 1 month (11/99; 11.1%), at 12 months (11/66; 16.7%), and in those with persistent depression (7/39; 17.9%).

**Table 2.**
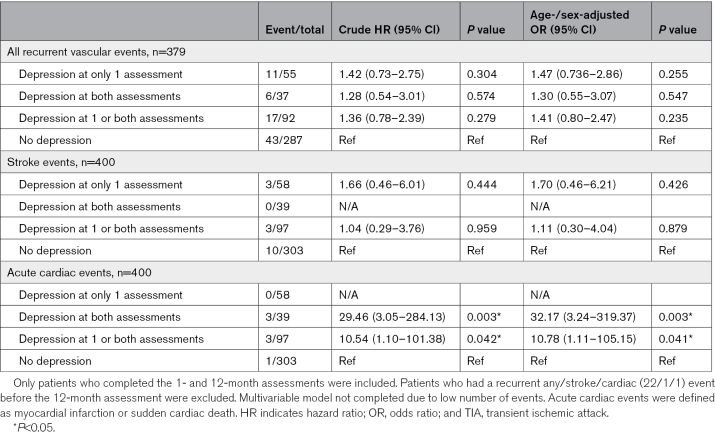
Crude and Age-/Sex-Adjusted Hazard Ratios for All Recurrent Acute Vascular Events, Stroke Events, and Acute Cardiac Events, Within 5 Years of Event, Stratified by the Presence of Depression at Only 1 Assessment, at Both Assessments, at 1 or Both Assessments, or No Depression After a TIA

Among survivors, persistent depression was also associated with increased disability over 5 years (adjusted odds ratio, 12.1 [95% CI, 6.8–23.7]; *P*<0.001), reduced QoL (coefficient, −0.245 [95% CI, −0.322 to −0.167]; *P*<0.001) and increased institutionalization (aHR, 5.83 [95% CI, 1.83–18.5]; *P*=0.003; Table 3).

**Table 3.**
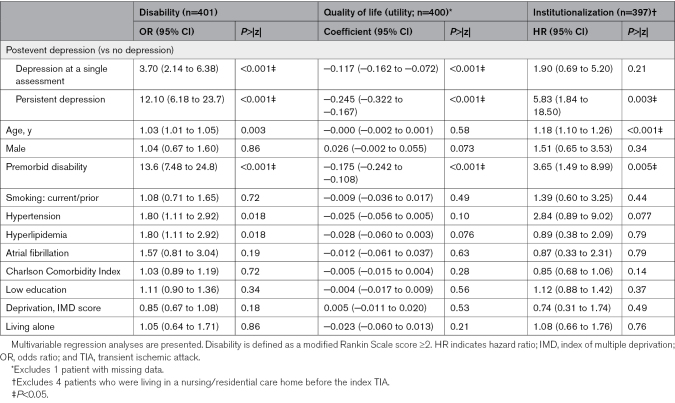
Associations for Disability, Health-Related Quality of Life, and Risk of Institutionalization at Follow-Up of Over 5 Years Stratified by the Presence of Depression at a Single Assessment, at Both Assessments (Persistent), or No Depression

## Discussion

In this study, we found that the prevalence of depression after a TIA was around 21% at 1 month, decreasing to 15% by 1 year, consistent with estimates from previous smaller studies that have used similar methodology.^30–32^ While a causal link between major stroke and an increased subsequent risk of depression is understandable and biologically plausible, it has been less clear whether the risk of depression is likely to be increased after a TIA or simply reflects pre-event levels.^1,2^ Our finding in OXVASC that the risk is highest at 1 month after a TIA and then declines significantly by 1 year shows that there is at least a temporal association.

It is less certain that there is a causal association between any TIA-associated brain injury or response to transient ischemia and risk of depression. We found no association with the duration of neurological symptoms or the presence of an acute lesion on brain imaging. If a causal association exists, it could be mediated via the stress of experiencing the neurological symptoms, anxiety about a major stroke, changes in lifestyle, which are advised, or side effects of new medications. There might also be an increased susceptibility to depression induced by underlying risk factors for cerebrovascular disease, such as hypertension, which are associated with depression in the absence of any cerebrovascular event.^15^

Irrespective of the mechanism(s) of any possible causal association between TIA and depression, it is important for clinicians to recognize and manage depression. Depression impairs QoL,^1^ reduces compliance with medication,^33^ and limits engagement with lifestyle changes, such as exercise and smoking cessation.^34^ Depression has also been shown to be associated with increased long-term mortality in the general population,^35^ as well as after stroke.^36,37^ Thus, although an association between post-TIA depression and increased mortality has not been assessed in previous studies, and the association in OXVASC was based on a relatively small number of events, the finding is not unexpected. Depression is associated with immune dysfunction, which may explain the association with increased risk of infections and progression of cancers,^12^ which accounted for several of the excess deaths in our study.

Our findings have some clinical implications. First, clinicians should be aware of the risk factors for depression within 12 months of a TIA: a history of depression, younger age, self-reporting a low mood at the initial assessment, disability, multimorbidity, living alone, and socioeconomic deprivation. Second, we found that depression at 1-month follow-up that resolved before 12 months was not associated with increased risk of death or recurrent stroke although statistical power was limited. Given the potential for early depression to resolve without treatment in a proportion of patients, it is uncertain which patients might benefit from early intervention versus rereview to detect persistence. Larger studies would be required to determine the predictors of persistence in patients with early depression. Third, if it is decided that treatment is required for a patient with depression after a TIA, it is uncertain what approach is likely to be most effective. Both antidepressant medication and psychological therapies for poststroke depression have been found to be associated with improved survival^38,39^ although evidence is more mixed in patients with cardiac disease.^40^ Any possible benefits of antidepressant medication have also to be balanced against the increased frequency of adverse effects, particularly in older, frail adults, such as orthostatic hypotension, falls, hyponatremia, arrhythmias, and delirium.^41^ In the absence of a large clinical trial designed to address this issue in patients with TIA or minor stroke, clinicians will have to rely on their clinical judgement. Finally, the responsibility for the detection and management of post-TIA depression remains unclear. A TIA or a stroke is a diagnosis often made in secondary care but with ongoing management delivered by primary care and no national guidelines to advise on screening for postevent psychological disorders. This lack of clarity on process and responsibility perhaps explains the low rate of prescription of antidepressants in our study and other reports^7^ although some patients may have been referred for psychological therapies, such as cognitive behavioral therapy, which are first-line for mild to moderate episodes of depression.^42^

Our study has some strengths, including the high participation rate, making it less vulnerable to selection bias and increasing generalizability, the face-to-face assessment, the use of a widely validated screening tool for depression, and long-term follow-up. However, there are many limitations. First, although the inclusion of OXVASC patients in the depression substudy was high, we cannot exclude a degree of participation bias. Second, as we did not interview the patients before their TIA, it is possible that we subsequently captured cases of previously undiagnosed pre-event depression. Third, we recorded the use of antidepressant medication but not psychological therapies or patients’ views on which aspects of their illness experience might have impacted their mood. Fourth, we did not assess the contribution of new medication to the risk of depression, but almost all patients had at least 1 medication change. Fifth, our follow-up spanned the COVID-19 pandemic, which may have impacted rates of depression, although we found no evidence of this, and none of the deaths during follow-up were attributed to COVID. Sixth, although mortality was assessed over 5 years, the association with depression could still be partly explained by occult pre-TIA pathology, and as our descriptor for persistent depression required an assessment at 12 months, the association is unknown for patients who died within that first year. However, previous studies in younger general population cohorts, or with longer follow-up, have consistently reported increased mortality in people with depression,^43^ and there is a mechanistic basis for the link.^44^ Finally, in common with previous studies, our assessment of depression was not based on a formal assessment by psychiatric services, but the GDS has been shown to be a sensitive screening tool, particularly in older adults.^22,45^

In conclusion, depression affects about 1-in-4 patients in the first year after a TIA, particularly those with known risk factors, and persistent depression is strongly associated with adverse outcomes, including all-cause mortality, apparently independent of acute ischemia on brain imaging.

## ARTICLE INFORMATION

### Acknowledgments

The authors thank all the staff in the general practices who collaborated in the OXVASC (Oxford Vascular Study).

### Sources of Funding

The OXVASC (Oxford Vascular Study) is supported by grants to Professor Rothwell from the National Institute for Health and Care Research Oxford Biomedical Research Center (IS-BRC-1215-20008), the Wolfson Foundation, the Wellcome Trust grant 104040/Z/14/Z, and the Masonic Charitable Foundation.

### Disclosures

Professor Rothwell received compensation from Abbott Vascular for consultant services. The other authors report no conflicts.

### Supplemental Material

Tables S1–S6

Figures S1–S2

STROBE Checklist

## Supplementary Material
