# Supplementary material for: Prevalence, Predictors, and Prognosis of Depression After Transient Ischemic Attack: A Population-Based Study
Source: Stroke. 2025 Nov 20;57(1):125–33. doi: 10.1161/STROKEAHA.125.052251 (PMC12721673; doi:10.1161/STROKEAHA.125.052251)

SUPPLEMENTARY MATERIAL

Contents

Table S1: Reasons why patients did not complete the 1-month or 12- month follow up assessments

Table S2: Comparison of the patients that completed no follow-up versus 1-month of follow up, and those that completed both 1 and 12-month versus those that completed 1 but not 12 month follow up

Table S3: Associations between baseline characteristics of TIA patients and their risk of persistent depression over 12-months (n=401).

Table S4: Associations between baseline characteristics of TIA patients and their risk of depression within the subsequent 12-months for patients who did not report low mood at baseline or have a past history of depression (n=324).

Table S5: Causes of death

Table S6: Crude, age / sex adjusted and multivariable adjusted hazard ratios for death within 5 years of event, stratified by presence of depression at a single assessment, at both assessments (persistent), depression present at the 1- and/or 12- month assessment, or no depression after a TIA for (a) all patients (n=401), (b) excluding those on anti-depressant treatment at either 0-, 1-, or 12-months (n=371), (c) excluding those with a past history of depression (n=290) and (d) excluding those with an acute lesion on brain imaging (n=350)

Figure S1: Flow chart of number of patients that completed follow-up at each time point

Figure S2: Frequency histogram of the distribution of the GDS scores at 1-month and 12-months for the 401 participants that completed both assessments.

Table S1: Reasons why patients did not complete the 1-month or 12- month follow up assessments. 84/562 patients (14.9%) did not complete the 1-month assessment and 77/478 patients (16.1%) completed the 1-month did not complete the 12-months assessment.

Reason	Reason for not completing 1-month assessment<br>n (% of total eligible)	Reason for not completing 12-months assessment<br>n (% of total eligible)
Completed assessment	*478 (85.0)*	*401 (71.4)*
Reason not given	17 (3.0)	25 (4.4)
Patient refused	15 (2.7)	5 (0.9)
Withdrew from active follow-up	14 (2.5)	20 (3.6)
Time constraints	14 (2.5)	1 (0.2)
Felt to be inappropriate (e.g. advanced dementia)	8 (1.4)	2 (0.4)
Lost contact	6 (1.1)	8 (1.4)
Language issue	6 (1.1)	2 (0.4)
Other (e.g. recent bereavement, too unwell)	4 (0.7)	4 (0.7)
Died prior to follow-up	-	9 (1.6)
COVID restrictions	-	1 (0.2)
**Total**	**84**	**77**

Table S2: Comparison of the patients that completed no follow-up versus 1-month of follow up, and those that completed both 1 and 12-month versus those that completed 1 but not 12 month follow up. p value for difference between completing *1 month of follow up and no follow-up and **all follow up and only 1 month of follow-up.

	Completed 1-month follow-up	Did not complete 1-month follow-up	p*	Completed 1 and 12-months follow-up	Completed 1 but not 12-months follow-up	p **
**Overall, n (% of total eligible)**	478 (85.1)	84 (14.9)	n/a	401 (71.4)	77 (13.7)	n/a
**Age at event, mean (s.d)**	70.4 (13.1)	73.1 (15.2)	0.096	70.3 (12.6)	71.1 (15.4)	0.651
**Female sex, n (%)**	239 (50.0)	52 (61.9)	**0.044**	198 (49.4)	41 (53.2)	0.534
**Past history of depression, n (%)[1]**	117 (25.2)	27 (34.2)	0.095	99 (25.4)	18 (24.0)	0.791
**Deprivation, mean IMD (s.d)**	10.3 (6.6)	10.9 (7.6)	0.459	10.1 (6.2)	11.6 (8.4)	0.068
**Charlson comorbidity index (s.d)**	1.1 (1.5)	1.4 (1.9)	0.114	1.1 (1.5)	1.3 (1.8)	0.298
**Diabetes mellitus, n (%)**	53 (11.1)	12 (14.3)	0.398	40 (10.0)	13 (16.9)	0.077
**Peripheral vascular disease, n (%)**	17 (3.6)	6 (7.1)	0.126	14 (3.5)	3 (3.9)	0.861
**Ischaemic heart disease, n (%)**	55 (11.5)	13 (15.5)	0.304	44 (11.0)	11 (14.3)	0.404
**Hypertension, n (%)**	263 (55.0)	51 (60.7)	0.332	215 (53.6)	48 (62.3)	0.159
**Hyperlipidaemia, n (%)**	154 (32.2)	26 (31.0)	0.819	125 (31.2)	29 (37.7)	0.264
**Atrial fibrillation, n (%)**	52 (10.9)	14 (16.7)	0.129	38 (9.5)	14 (18.2)	**0.025**
**Smoking – current/prior, n (%)[2]**	238 (49.9)	44 (52.4)	0.674	200 (49.9)	38 (50.0)	0.984
**Lives alone (%)**	118 (24.7)	23 (27.4)	0.599	96 (23.9)	22 (28.6)	0.388
**Premorbid modified Rankin Score ≥ 2 (%)**	76 (15.9)	28 (33.3)	**<0.001**	53 (13.2)	23 (29.9)	**<0.001**
**Education ≤12 years, n (%)[3]**	241 (51.2)	48 (57.8)	0.262	196 (49.2)	45 (60.8)	0.071
**ABCD2 score≥4, n (%)[4]**	256 (54.0)	39 (47.6)	0.280	216 (54.7)	41 (54.7)	0.901
**Moderate-to-severe WMD (%)[5]**	146 (30.8)	25 (30.9)	0.991	125 (31.3)	21 (28.0)	0.567
**Acute lesion on brain imaging (%)[5]**	63 (13.3)	10 (12.2)	0.791	50 (12.5)	13 (17.3)	0.257
**Depression at 1 month, n (%)**	n/a	n/a	n/a	79 (19.7)	20 (26.0)	0.213

Number of patients with missing data for a specified variable: [1]19, [2]1, [3]8, [4]6 [5]7. IMD = Index of Multiple Deprivation, WMD = white matter disease.

Table S3: Associations between baseline characteristics of TIA patients and their risk of persistent depression over 12-months (n=401). Age/sex adjusted odds ratio and multivariable regression analysis also included. The odds ratios for age are calculated per 10 year increase and for deprivation per standard deviation (s.d) increase. Depression at 1-month and recurrent events were not included in the model.

**Baseline variable**	**All**	**Persistent depression**	**No Depression**	**Age/sex-adj OR (95% CI)**	**p**	**Multivariable[#] OR (95% CI)**	**p**
Female (%)	198 (50.6)	18 (46.2)	180 (49.7)	0.88 (0.45-1.71)	0.708	0.65 (0.28-1.47)	0.298
Mean age (s.d)	70.3 (12.6)	69.3 (15.4)	70.4 (12.3)	0.93 (0.72-1.21)	0.602	0.66 (0.74-0.91)	**0.011**
Low mood at baseline (%)*[1]	59 (15.0)	18 (48.6)	41 (11.5)	7.21 (3.50-14.88)	**<0.001**	5.47 (2.35-12.75)	**<0.001**
Past history of depression (%)*[2]	99 (25.4)	17 (44.7)	82 (23.4)	2.69 (1.35-5.37)	**0.005**	1.77 (0.77-4.04)	0.179
ABCD2 ≥ 4 (%)*[3]	215 (53.9)	22 (57.9)	193 (53.3)	1.30 (0.64-2.63)	0.476	-	
Symptoms > 1 hour (%)*[4]	166 (41.6)	15 (38.5)	193 (53.3)	0.86 (0.44-1.70)	0.662	-	
Smoking – current/prior (%)	200 (49.9)	22 (56.4)	178 (49.2)	1.32 (0.67-2.60)	0.425	-	
Charlson Comorbidity Index (s.d)	1.1 (1.5)	1.5 (1.9)	1.0 (1.4)	1.22 (1.00-1.50)	0.051	-	
Diabetes mellitus	40 (10.0)	3 (7.7)	37 (10.2)	0.72 (0.21-2.46)	0.599	-	
Peripheral vascular disease	14 (3.5)	2 (5.1)	12 (3.3)	1.66 (0.34-7.96)	0.530	-	
Ischaemic heart disease	44 (11.0)	9 (23.1)	35 (9.7)	3.13 (1.32-7.43)	**0.010**	3.07 (1.09-8.63)	**0.034**
Hypertension	215 (53.6)	19 (48.7)	196 (54.1)	0.84 (0.41-1.72)	0.636	-	
Hyperlipidaemia	125 (31.2)	12 (30.8)	113 (31.2)	0.99 (0.48-2.06)	0.979	-	
Atrial fibrillation	38 (9.5)	5 (12.8)	33 (9.1)	1.54 (0.56-4.29)	0.406	-	
Lives alone (%)	96 (23.9)	13 (33.3)	83 (22.9)	1.93 (0.91-4.10)	0.086	1.39 (0.56-3.42)	0.474
Modified Rankin score ≥ 2 (%)	53 (13.2)	15 (38.5)	38 (10.5)	8.24 (3.56-19.09)	**<0.001**	9.71 (3.63-26.03)	**<0.001**
Low education (%)*[5]	196 (49.4)	24 (61.5)	172 (47.5)	1.79 (0.90-3.53)	0.096	1.35 (0.61-2.98)	0.463
Deprivation, mean IMD (s.d)	10.1 (6.2)	12.2 (6.0)	9.8 (6.2)	1.37 (1.03-1.82)	**0.030**	1.43 (0.98-2.08)	0.061
Moderate-to-severe WMD (%)*[6]	125 (31.3)	14 (36.8)	111 (30.7)	1.50 (0.70-3.20)	0.294	-	
Acute lesion on imaging (%)*[6]	50 (12.5)	4 (10.5)	46 (12.7)	0.79 (0.27-2.36)	0.683	-	

*Number of patients with missing data for a specified variable: [1]7, [2]12, [3]2, [4]5, [5]4, [6]1.IMD = Index of Multiple Deprivation, WMD = white matter disease. . [#]Only variables that were found to be p<0.1 in the age/sex adjusted analysis were included in the multivariable regression analysis. Age/sex were also included as they have been found to be associated with depression in prior studies. [$]Low mood at baseline is defined as the patient stating yes when asked if they "often feel sad or depressed."

Table S4: Associations between baseline characteristics of TIA patients and their risk of depression within the subsequent 12-months for patients who did not report low mood at baseline or have a past history of depression (n=324). Age/sex adjusted odds ratio and multivariable regression analysis for the risk of depression within 12-month also included. The odds ratios for age are calculated per 10 year increase and for deprivation per standard deviation (s.d) increase

Baseline variable	All	Depression Yes	No	Age/sex-adj OR (95% CI)	p	Multivariable[#] OR (95% CI)	p
Female (%)	159 (49.1)	30 (56.6)	129 (47.6)	1.41 (0.77-2.58)	0.260	1.32 (0.66-2.64)	0.428
Mean age (s.d)	71.4 (13.0)	72.3 (18.0)	71.3 (11.8)	1.00 (0.82-1.34)	0.756	0.66 (0.48-0.90)	**0.004**
ABCD2 ≥ 4 (%)*[1]	176 (54.8)	30 (56.6)	146 (53.9)	1.05 (0.57-1.95)	0.869	-	
Symptoms > 1 hour (%)*[2]	120 (37.5)	20 (37.7)	100 (37.5)	1.04 (0.57-1.92)	0.898	-	
Smoking – current/prior (%)*[3]	148 (45.7)	22 (41.5)	126 (46.7)	0.90 (0.48-1.71)	0.751	-	
Charlson Comorbidity Index (s.d)	1.2 (1.6)	1.8 (1.8)	1.1 (1.6)	1.27 (1.07-1.50)	**0.007**	1.23 (1.01-1.48)	**0.039**
Diabetes mellitus (%)	35 (10.8)	6 (11.3)	29 (10.7)	1.11 (0.44-2.85)	0.823	-	
Peripheral vascular disease (%)	11 (3.4)	0 (0)	11 (4.1)	0.00	0.999	-	
Ischaemic heart disease (%)	34 (10.5)	8 (15.1)	26 (9.6)	1.84 (0.75-4.50)	0.180	-	
Hypertension (%)	177 (54.6)	30 (56.6)	147 (54.2)	1.05 (0.55-1.99)	0.880	-	
Hyperlipidaemia (%)	101 (31.2)	15 (28.3)	86 (31.7)	0.88 (0.45-1.71)	0.704	-	
Atrial fibrillation (%)	36 (11.1)	11 (20.8)	25 (9.2)	2.65 (1.17-5.981)	**0.019**	1.44 (0.55-3.75)	0.233
Lives alone (%)	74 (22.8)	18 (34.0)	56 (20.7)	1.88 (0.95-3.75)	0.072	1.61 (0.74-3.49)	0.233
modified Rankin Score ≥ 2 (%)	48 (14.8)	20 (37.7)	28 (10.3)	6.69 (3.08-14.53)	**<0.001**	5.98 (2.52-14.19)	**<0.001**
Low education (%)*[4]	160 (50.2)	27 (51.9)	133 (49.8)	1.10 (0.60-2.00)	0.760	-	
Deprivation, mean IMD (s.d)	10.1 (6.8)	11.9 (8.0)	9.7 (6.5)	1.32 (1.03-1.71)	**0.030**	1.36 (1.03-1.79)	**0.030**
Moderate-to-severe WMD (%)*[5]	104 (32.4)	23 (45.1)	81 (30.0)	2.07 (1.06-4.02)	**0.033**	2.31 (1.10-4.85)	**0.026**
Acute lesion on brain imaging (%)*[5]	47 (14.6)	7 (13.7)	40 (14.8)	0.93 (0.39-2.21)	0.863	-	

*Number of patients with missing data for a specified variable: all: [1]11, [2]17, [3]5, [4]5 [5]1, [6]7, [7]5, excluding low mood: [1]3, [2]4, [3]1, [4]5 [5]3. IMD = Index of Multiple Deprivation, WMD = white matter disease. [#]Only variables that were found to be p<0.1 in the age/sex adjusted analysis were included in the multivariable regression analysis. Age/sex were also included as they have been found to be associated with depression in prior studies.

Table S5: Causes of death Patients that died prior to the 12-month assessment, or failed to complete both the 1- and 12-month assessments were excluded. No deaths were attributed to suicide or COVID-19.

**Cause**	**All deaths**	**No depression – deaths**	**Single point depression – deaths**	**Persistent depression - deaths**
	**N (%)**	**N (%)**	**N (%)**	**N (%)**
Sepsis / pneumonia	11 (22.0)	7 (25.0)	0 (0)	4 (30.8)
Malignancy	9 (18.0)	6 (21.4)	2 (22.2)	1 (7.7)
Old age	5 (10.0)	3 (10.7)	1 (11.1)	1 (7.7)
Acute vascular death	5 (10.0)	2 (7.1)	1 (11.1)	2 (15.4)
Neurodegenerative	4 (8.0)	2 (7.1)	0 (0)	2 (15.4)
Heart failure	4 (8.0)	2 (7.1)	1 (11.1)	1 (7.7)
Other*	2 (4.0)	2 (7.1)	0 (0)	0 (0)
Trauma	1 (2.0)	0 (0)	0 (0)	1 (7.7)
Cause of death awaited	9 (18.0)	4 (14.3)	4 (44.4)	1 (7.7)
**Total**	**50**	**28**	**9**	**13**

Other includes decompensated liver disease, pancreatitis*

Table S6: Crude, age / sex adjusted and multivariable adjusted hazard ratios for death within 5 years of event, stratified by presence of depression at a single assessment, at both assessments (persistent), depression present at the 1- and/or 12- month assessment, or no depression after a TIA for (a) all patients (n=401), (b) excluding those on anti-depressant treatment at either 0-, 1-, or 12-months (n=371), (c) excluding those with a past history of depression (n=290) and (d) excluding those with an acute lesion on brain imaging (n=350)
Patients that died prior to the 12-month assessment were excluded. Multivariable model includes: age, sex, charlson comorbidity index, modified Rankin score, smoking history, low education status, socioeconomic deprivation, history of hypertension, hyperlipidaemia, atrial fibrillation and living alone. status.

	Event / total	Crude HR (95% CI)	p	Age/sex-adj OR (95% CI)	p	Multivariable HR (95% CI)	p
**(a) Total cohort**							
Depression at a single assessment	9/58	1.73 (0.82-3.67)	0.152	1.69 (0.79-3.60)	0.177	1.39 (0.61-3.15)	0.431
Persistent depression	13/39	4.29 (2.22-8.29)	**<0.001**	5.46 (2.78-10.70)	**<0.001**	4.58 (2.07-10.13)	**<0.001**
Depression at 1- and/or 12-month assessments	26/97	2.62 (1.53-4.67)	**<0.001**	2.84 (1.61-5.01)	**<0.001**	2.27 (1.21-4.27)	**0.011**
No depression	28/304	ref	ref	ref	ref	ref	ref
**(b) Excluding on anti-depressant treatment**							
Depression at a single assessment	8/54	1.89 (0.84-4.21)	0.120	1.74 (0.77-3.93)	0.187	1.39 (0.57-3.41)	0.468
Persistent depression	11/32	5.05 (2.46-10.37)	**<0.001**	6.16 (2.92-12.98)	**<0.001**	5.39 (2.18-13.32)	**<0.001**
Depression at 1- and/or 12-month assessments	23/86	2.96 (1.61-5.43)	**<0.001**	2.94 (1.59-5.47)	**0.001**	2.29 (1.13-4.63)	**0.022**
No depression	23/285	ref	ref	ref	ref	ref	ref
**(c) Excluding past history of depression**							
Depression at a single assessment	8/34	2.15 (0.86-5.34)	0.101	2.04 (0.81-5.11)	0.130	1.68 (0.61-4.67)	0.319
Persistent depression	11/21	7.10 (3.32-15.21)	**<0.001**	7.03 (3.20-15.44)	**<0.001**	8.03 (2.95-21.85)	**<0.001**
Depression at 1- and/or 12-month assessments	19/55	3.80 (1.97-7.34)	**<0.001**	3.62 (1.85-7.07)	**<0.001**	3.17 (1.51-6.68)	**0.002**
No depression	31/235	ref	ref	ref	ref	ref	ref
**(d) Excluding acute lesion on brain imaging**							
Depression at a single assessment	11/53	1.70 (0.76-3.77)	0.196	1.65 (0.74-3.70)	0.223	1.34 (0.55-3.23)	0.516
Persistent depression	12/34	4.29 (2.10-8.77)	**<0.001**	5.41 (2.60-11.26)	**<0.001**	4.62 (1.94-11.01)	**0.001**
Depression at 1- and/or 12-month assessments	23/87	2.61 (1.43-4.76)	**0.002**	2.75 (1.50-5.05)	**0.001**	2.18 (1.09-4.34)	**0.027**
No depression	24/263	ref	ref	ref	ref	ref	ref

Figure S1: Flow chart of number of patients that completed follow-up at each time point

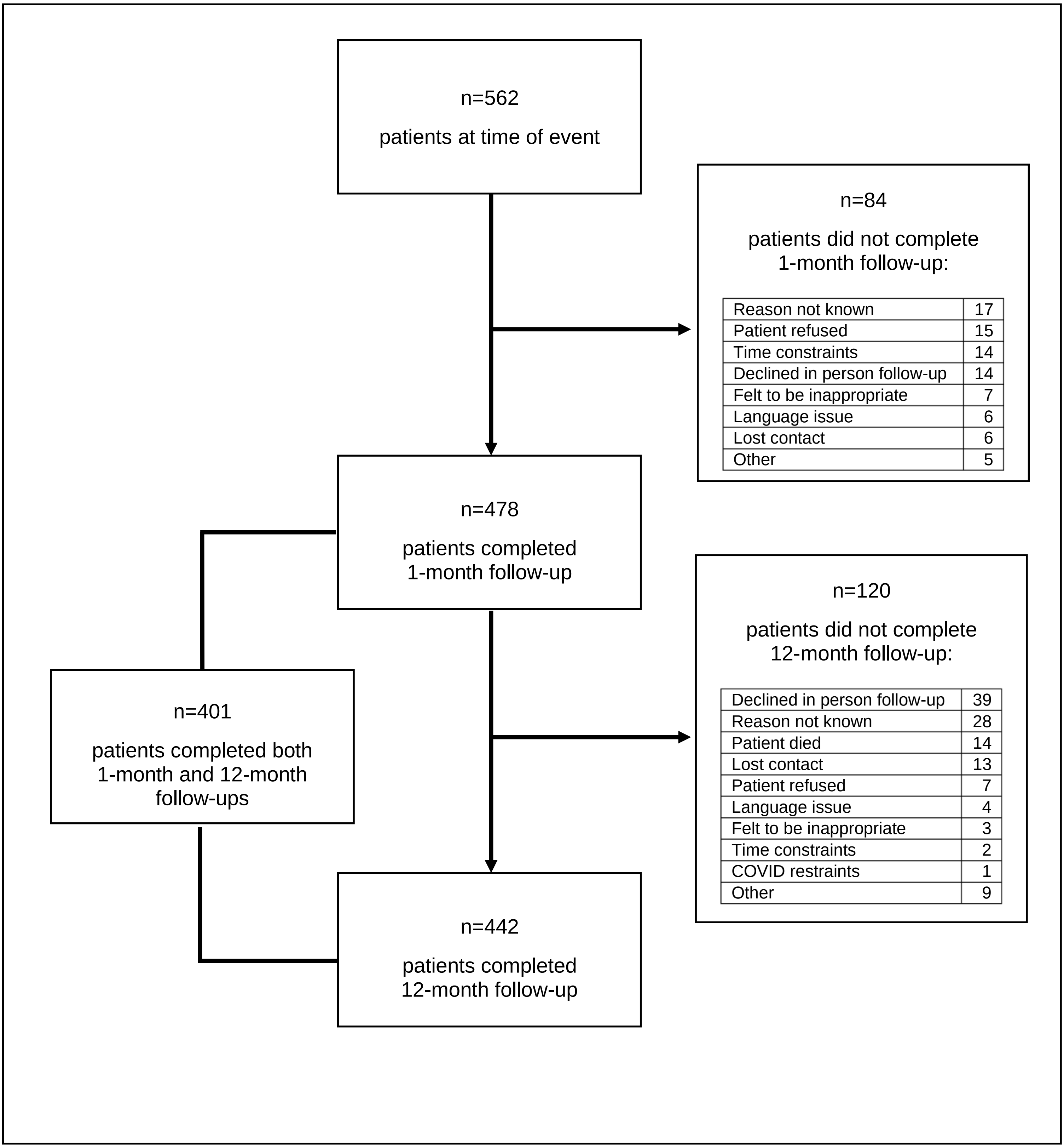

Figure S2: Frequency histogram of the distribution of the GDS scores at 1-month and 12-months for the 401 participants that completed both assessments.

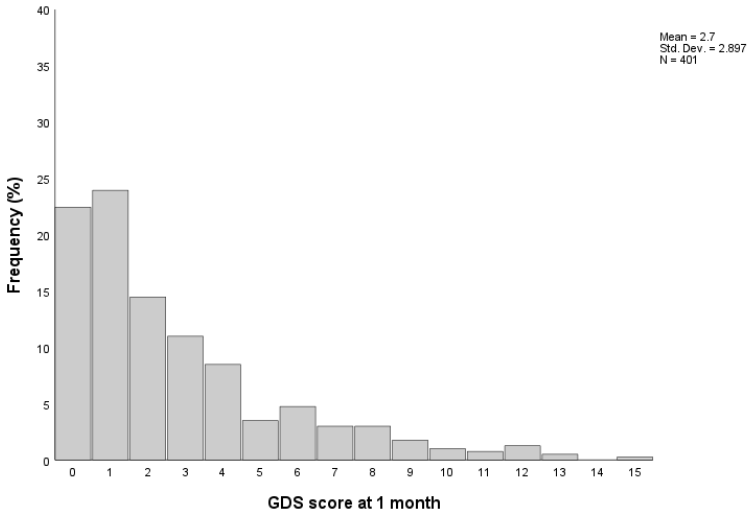

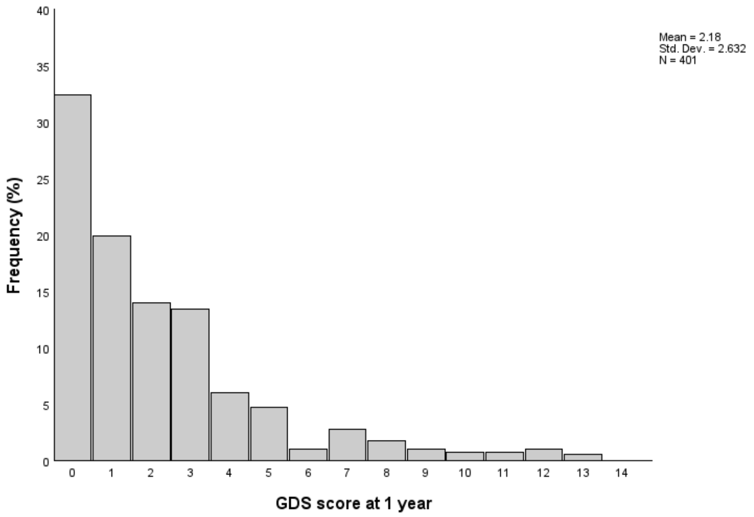

Supplement: Supplementary file 1 [file str-57-125-s001.pdf]
