# Supplementary material for: Prevalence, Predictors, and Prognosis of Depression After Transient Ischemic Attack: A Population-Based Study
Source: Stroke. 2025 Nov 20;57(1):125–33. doi: 10.1161/STROKEAHA.125.052251 (PMC12721673; doi:10.1161/STROKEAHA.125.052251)
Supplement: Supplementary file 2 [file str-57-125-s002.pdf]

STROBE Statement — Checklist of items for:

"Prevalence, predictors and prognosis of depression after transient ischaemic attack: a population-based study."

	Item No	Recommendation	Line number
**Title and abstract**	1	(*a*) Indicate the study's design with a commonly used term in the title or the abstract	1-2
		(*b*) Provide in the abstract an informative and balanced summary of what was done and what was found	26--50
**Introduction**			
Background/rationale	2	Explain the scientific background and rationale for the investigation being reported	52-55, 61-63
Objectives	3	State specific objectives, including any prespecified hypotheses	60-72
**Methods**			
Study design	4	Present key elements of study design early in the paper	74-88
Setting	5	Describe the setting, locations, and relevant dates, including periods of recruitment, exposure, follow-up, and data collection	77-93
Participants	6	(*a*) Give the eligibility criteria, and the sources and methods of selection of participants. Describe methods of follow-up	81-93,106
		(*b*) For matched studies, give matching criteria and number of exposed and unexposed	n/a
Variables	7	Clearly define all outcomes, exposures, predictors, potential confounders, and effect modifiers. Give diagnostic criteria, if applicable	94-114, 118-122
Data sources/ measurement	8*	For each variable of interest, give sources of data and details of methods of assessment (measurement). Describe comparability of assessment methods if there is more than one group	94-114, 118-122
Bias	9	Describe any efforts to address potential sources of bias	114
Study size	10	Explain how the study size was arrived at	77-79
Quantitative variables	11	Explain how quantitative variables were handled in the analyses. If applicable, describe which groupings were chosen and why	125-162
Statistical methods	12	(*a*) Describe all statistical methods, including those used to control for confounding	125,127-145,149-164
		(*b*) Describe any methods used to examine subgroups and interactions	134-135
		(*c*) Explain how missing data were addressed	In the subtext of Tables
		(*d*) If applicable, explain how loss to follow-up was addressed	126-127
		(*e*) Describe any sensitivity analyses	134-135
**Results**			
Participants	13*	(a) Report numbers of individuals at each stage of study—	169-170, Sup

		eg numbers potentially eligible, examined for eligibility, confirmed eligible, included in the study, completing follow-up, and analysed	Fig1
		(b) Give reasons for non-participation at each stage	Supplementary Tables 1 and 2, Fig1
		(c) Consider use of a flow diagram	Sup Fig1
Descriptive data	14*	(a) Give characteristics of study participants (eg demographic, clinical, social) and information on exposures and potential confounders	Table 1
		(b) Indicate number of participants with missing data for each variable of interest	Table 1 subtext
		(c) Summarise follow-up time (eg, average and total amount)	n/a
Outcome data	15*	Report numbers of outcome events or summary measures over time	177-181
Main results	16	(*a*) Give unadjusted estimates and, if applicable, confounder-adjusted estimates and their precision (eg, 95% confidence interval). Make clear which confounders were adjusted for and why they were included	177-181, 182-187, 191-196, 197-202,203-205,207, 208-219. Tables 1, 2. Supp Tables 3-5 & 7.
		(*b*) Report category boundaries when continuous variables were categorized	112, 146
		(*c*) If relevant, consider translating estimates of relative risk into absolute risk for a meaningful time period	n/a
Other analyses	17	Report other analyses done—eg analyses of subgroups and interactions, and sensitivity analyses	192-196
**Discussion**			
Key results	18	Summarise key results with reference to study objectives	221-223
Limitations	19	Discuss limitations of the study, taking into account sources of potential bias or imprecision. Discuss both direction and magnitude of any potential bias	272-288
Interpretation	20	Give a cautious overall interpretation of results considering objectives, limitations, multiplicity of analyses, results from similar studies, and other relevant evidence	289-291
Generalisability	21	Discuss the generalisability (external validity) of the study results	246-262
**Other information**			
Funding	22	Give the source of funding and the role of the funders for the present study and, if applicable, for the original study on which the present article is based	296-301
